# A Hybrid Machine Learning Framework for Interpretable Kinetics of α‐Tocopherol and Myricetin Synergism

**DOI:** 10.1111/1750-3841.71291

**Published:** 2026-07-14

**Authors:** Jiakai Lu, Sankaran Iyer, Ipek Bayram, Eric A. Decker, Shyamvanshikumar P. Singh, Carlos M. Corvalan

**Affiliations:** ^1^ Department of Food Science University of Massachusetts Amherst Massachusetts USA; ^2^ Transport Phenomena Laboratory Department of Food Science Purdue University West Lafayette Indiana USA; ^3^ Department of Food Engineering Faculty of Engineering Middle East Technical University Ankara Türkiye

## Abstract

Predicting the stabilizing efficacy of antioxidant mixtures in food oil emulsions is highly complex due to synergistic or antagonistic interactions between individual antioxidants. To address this challenge, we present an innovative hybrid machine learning framework, known as universal differential equations (UDEs), which integrates the expressive power of deep learning with the mechanistic boundaries of traditional kinetic models. We demonstrate the utility of this data‐efficient approach by characterizing the coupled degradation dynamics of α‐tocopherol in the presence of myricetin in oil. By embedding compact artificial neural networks directly into a system of ordinary differential equations, the hybrid UDE model successfully learned the hidden interactions from a small dataset, quantitatively revealing their mutualistic protection. Furthermore, we translated these machine‐learned interactions into an interpretable, fully analytical model based on Hill‐type saturation kinetics. Critically, this transparent analytical model not only accurately reproduced the training data (R^2^ = 0.998) but successfully extrapolated antioxidant dynamics to previously unseen experimental formulations (*R* ^2^ = 0.978) with a fivefold increase in myricetin concentration. This work provides a powerful, interpretable AI tool for understanding complex kinetic interactions in food systems, with broad applications for accelerating product development, optimizing preservation strategies, and extending food shelf life.

## 1 Introduction

Enhancing the stability and shelf life of emulsions remains a persistent challenge in food technology. Lipid oxidation, a primary driver of quality deterioration in oil emulsions, can be mitigated by incorporating food antioxidants (Hennebelle et al. 2024; Ghelichi et al. 2023; Villeneuve et al. 2023). However, the effectiveness of these interventions depends not only on individual antioxidant strength but also on the complex interplay between different antioxidants within a mixture (Berton‐Carabin and Villeneuve 2023; Hennebelle et al. 2024; Laguerre et al. 2007). Characterizing these interactions, which often exhibit synergistic (cooperative) or antagonistic (inhibitory) effects, is critical for optimizing antioxidant formulations and quantitatively predicting their impact on stability and shelf life (Bayram and Decker 2023).

Recent advances in artificial intelligence (AI) and machine learning (ML) provide powerful tools for analyzing complex datasets (Zhou et al. 2019; Murphy 2022; Krenn et al. 2022), offering a highly flexible, data‐driven approach to understanding interacting phenomena in food science. However, the direct application of pure deep learning to chemical kinetics is often limited by two major bottlenecks. First, traditional ML algorithms are highly data‐intensive, but high‐quality, time‐resolved experimental data in food chemistry is typically sparse and expensive to acquire. Second, neural networks function as “black boxes” that lack the mechanistic interpretability of traditional dynamical systems models (Minh et al. 2022; Murdoch et al. 2019). For food chemists and formulators, bridging this gap between data‐driven algorithms and mechanistic modeling is crucial for advancing predictive capabilities, trusting the model outputs, and gaining deeper chemical insights (Krenn et al. 2022; Raissi et al. 2019).

To overcome these bottlenecks, an emerging scientific machine learning framework known as universal differential equations (UDEs) offers a transformative, data‐efficient “gray‐box” approach (Rackauckas et al. 2020). UDEs uniquely integrate the data‐driven strengths of neural network models with the mechanistic structure of classical ordinary differential equation (ODE) models, creating a powerful hybrid framework for modeling complex systems (Philipps et al. 2025). This hybrid architecture directly addresses the “black box” limitation by allowing the known mechanistic behaviors of a system (e.g., standard baseline decay) to be modeled explicitly, using the neural network as a universal approximator to learn only the unknown interaction terms from the data (Giampiccolo et al. 2024; Rackauckas et al. 2020; Fulkerson et al. 2025). Simultaneously, constraining the neural network within a differential equation drastically narrows the admissible solution search space. By tasking the network with approximating only the unknown components of a dynamical system (rather than requiring it to learn the global behavior from a vast space of arbitrary potential functions), this approach effectively mitigates the challenge of data scarcity. Consequently, the framework is resistant to overfitting and capable of uncovering interpretable, relevant mechanisms from the sparse and noisy datasets typical of experimental food science (Philipps et al. 2025; de Rooij et al. 2025). Furthermore, unlike standard empirical curve fitting, the ODE structure grants the model robust extrapolation capabilities and the power to accurately forecast complex nonlinear dynamics well beyond its training domain (Kuwahara and Bauch 2024).

In this study, we assess the efficacy of this hybrid ML framework for food applications by analyzing experimental data on the degradation of alpha‐tocopherol in the presence of myricetin. This case study allows us to investigate the complex interplay between two common antioxidants (Bayram and Decker 2024; Parra‐Escudero et al. 2025). Our machine learning‐augmented approach successfully uncovers their mutualistic interaction from a limited dataset. Furthermore, to gain actionable mechanistic understanding for food formulation, we translated the machine‐learned interaction terms into an interpretable, fully analytical reduced‐order model based on Hill‐type saturation kinetics (Hill 1910; Alon 2019). We demonstrate that this transparent analytical model not only provides valuable insight into the underlying degradation dynamics but accurately extrapolates and predicts antioxidant behavior in an entirely new, unseen formulation scenario.

## 2 Materials and Methods

### 2.1 Experimental Characterization of Antioxidant Degradation

The experimental data used to train and validate our hybrid machine learning model were obtained from our previously published degradation assays. Detailed procedures regarding sample preparation, accelerated oxidation conditions, and analytical quantification have been thoroughly described elsewhere (Bayram and Decker 2024; Parra‐Escudero et al. 2025).

Briefly, to study antioxidant interactions without interference from endogenous compounds, commercial soybean oil was stripped of natural antioxidants and pro‐oxidants using column chromatography with silicic acid and activated charcoal. Oxidation was monitored in a 1:1 mixture of the stripped soybean oil and medium‐chain triacylglycerols. This clean matrix was fortified with a constant 50 µM of α‐tocopherol and varying initial concentrations of myricetin (e.g., initial myricetin:tocopherol molar ratios of 2:10 and 10:10). Samples were subjected to accelerated oxidation by incubation at 60°C in the dark. At specified time intervals, the degradation trajectories of both α‐tocopherol and myricetin were quantified via high‐performance liquid chromatography (HPLC) equipped with fluorescence and photodiode array detection. For the 2:10 myricetin:tocopherol ratio, myricetin and α‐tocopherol concentrations were measured at 12 and 18 discrete time points, respectively. For the 10:10 ratio, myricetin and α‐tocopherol concentrations were measured at 18 and 28 time points, respectively. All measurements were performed in triplicate at each sampling point. Experimental mean values and variability are presented throughout the manuscript using symbols and error bars in the corresponding figures.

### 2.2 Hybrid Deep Learning Model (Universal Differential Equations)

The central modeling challenge in complex food emulsions is that the exact nature of antioxidant interactions (synergistic, antagonistic, or concentration‐dependent) is frequently unknown and highly nonlinear. To uncover the coupled dynamics between α‐tocopherol and myricetin without forcing the data into preconceived kinetic models, we developed a machine learning‐augmented framework.

Based on the universal differential equations (UDE) framework (Giampiccolo et al. 2024; Rackauckas et al. 2020), this hybrid approach embeds artificial neural networks directly into a traditional system of kinetic differential equations. This allows us to separate the standard, linear baseline degradation of the antioxidants from their unknown interaction behaviors. The model is mathematically expressed as:

(1a) $$du/dt\;=-a_{1}\;u+N_{1}\left(u,v\right)\;u$$

(1b) $$dv/dt=-a_{2}\;v+N_{2}\left(u,v\right)v$$

Here, *u* and *v* represent the transient concentrations of α‐tocopherol and myricetin, respectively. The terms *a1* and *a2* are traditional linear decay rate coefficients. The unknown, complex interaction effects between the two antioxidants are represented by *N1* and *N2*, which are deep neural networks. By the universal approximation theorem (Kratsios 2021), these networks act as *discovery functions*, capable of approximating any continuous mathematical relationship governing how the presence of myricetin (*v*) alters the degradation rate of tocopherol (*u*), and vice versa. Inputs to the UDE consisted of time resolved concentrations *u*(*t*) and *v*(*t*).

The neural networks *N1* and *N2* were implemented as fully connected, feed‐forward architectures using the TensorFlow library (Abadi et al. 2015). To prevent the model from overfitting on the relatively sparse datasets typical of food chemistry experiments, the networks were kept intentionally compact, consisting of just two hidden layers with 10 neurons each.

Importantly, the hyperbolic tangent (*tanh*) activation function was used for the hidden layers. In purely data‐driven machine learning, unbounded activation functions are common; however, in chemical kinetics, interaction effects are physically bounded (e.g., they eventually plateau due to substrate depletion or phase saturation). The bounded, sigmoidal nature of the *tanh* function naturally enforces these physical constraints, ensuring the neural network predicts chemically plausible saturation kinetics rather than, for example, values growing to infinity (Murphy 2022).

#### 2.2.1 Model Training and Optimization

**FIGURE 1:**
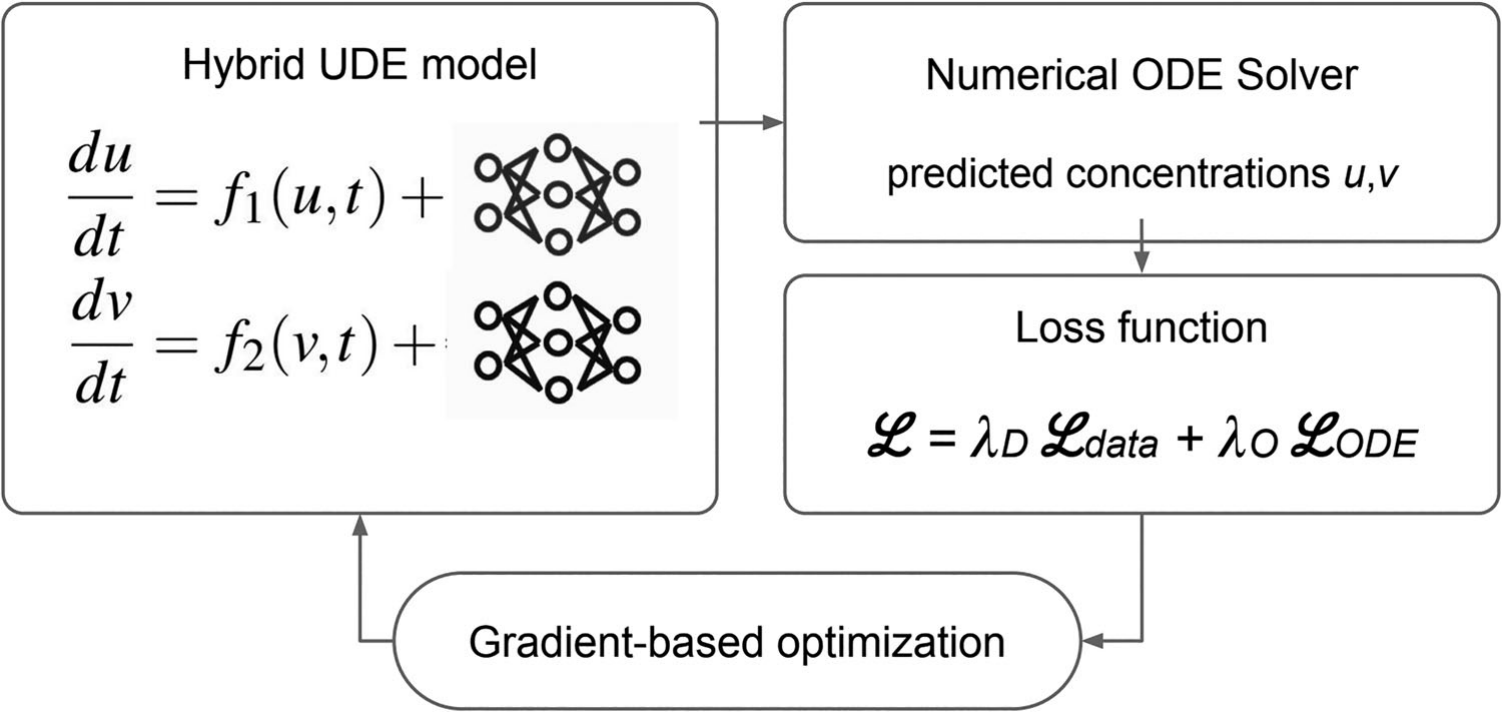
Sketch of the universal differential equation (UDE) framework. The hybrid UDE model combines known terms (*f1*, *f2*) with deep neural networks to represent the unknown antioxidant interactions. A numerical ODE solver integrates the dynamic system to generate predicted concentrations (*u*, *v*). These predictions are then evaluated using a composite loss function ℒ, which penalizes discrepancies with both the experimental data and the governing ODEs. Finally, a gradient‐based optimization algorithm iteratively updates the neural network weights and kinetic parameters to minimize the loss, closing the training loop.

A key advantage of the UDE approach is the simultaneous optimization of all unknown parameters from a single dataset: both the neural network weights and the kinetic decay coefficients (*a1*, *a2*). The training process iteratively solved the hybrid differential equation system (Equation 1) to generate predicted concentration trajectories over time. The mean squared error (MSE) between these simulated trajectories and the actual experimental HPLC data (along with its derivative) was computed. This error was then backpropagated through the entire system, including the ODE solver, to continuously update and refine the parameters. This end‐to‐end optimization was performed using the Adam algorithm (Murphy 2022), which is well‐suited for this task due to its adaptive learning rate and robust performance in converging with small dataset on the underlying chemical dynamics. A sketch of this iterative machine learning pipeline, from the hybrid system formulation and numerical integration to the gradient‐based parameter optimization, is shown in Figure 1.

To ensure the hybrid model learned the true underlying chemical kinetics rather than simply memorizing experimental noise (overfitting), the neural networks were evaluated using a *k*‐fold cross‐validation. Specifically, the experimental dataset was partitioned using fivefold cross‐validation, and the model was iteratively trained on 80% of the data and validated on the remaining 20%.

## 3 Results and Discussion

**FIGURE 2:**
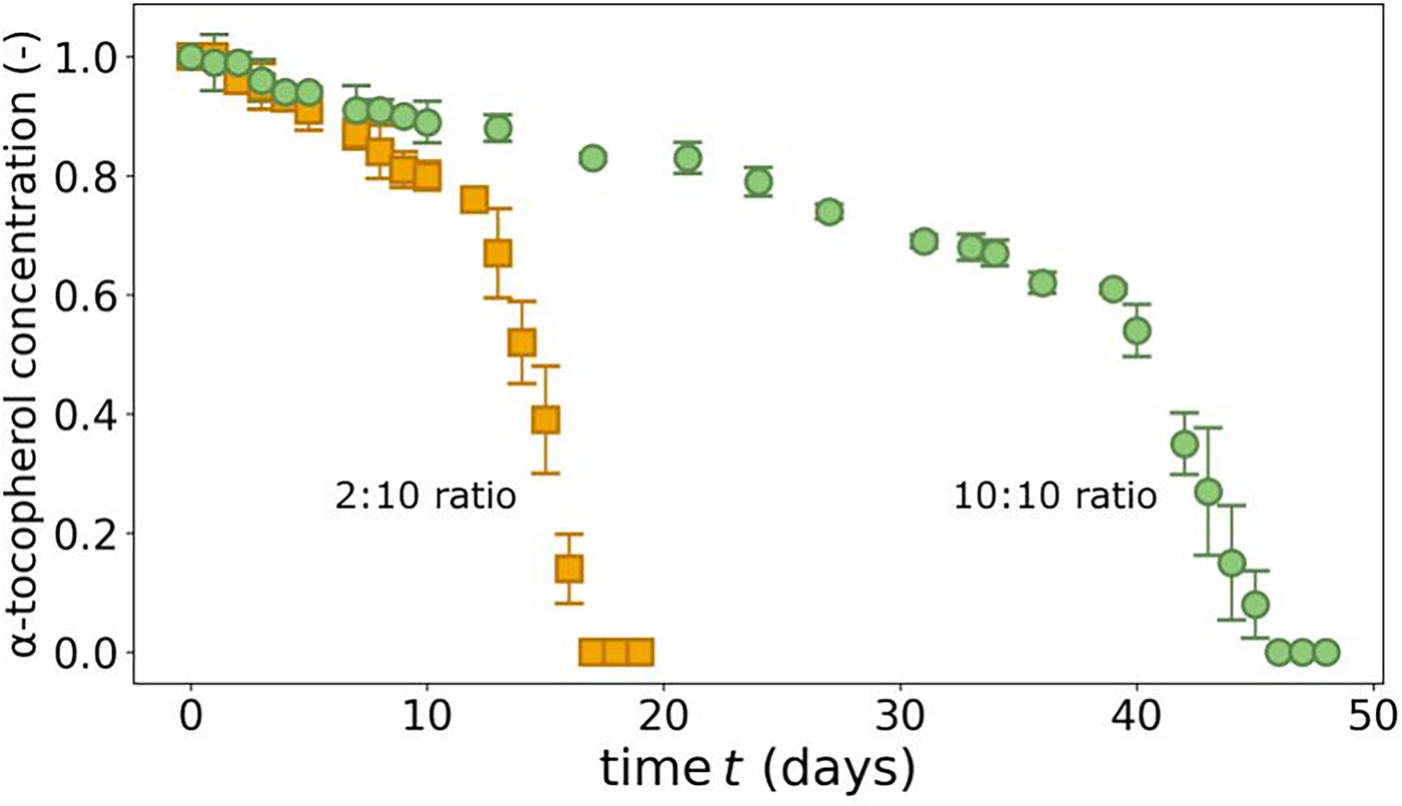
Myricetin extends tocopherol shelf life. Symbols represent experimentally measured values of the normalized concentration of α‐tocopherol remaining over time for two initial myricetin:tocopherol ratios: 2:10 (orange) and 10:10 (green). The degradation is distinctly biphasic, consistent with an initial slow saturation phase where myricetin offers effective protection, followed by a rapid decay. Error bars represent the standard deviation of three replicates. Normalized concentrations were calculated by dividing the α‐tocopherol concentration by its initial concentration of 50 µM.

Experimental results demonstrate that the shelf life of α‐tocopherol in oil is highly dependent on the initial concentration of myricetin in the mixture (Figure 2). Specifically, increasing the initial myricetin:tocopherol molar ratio from 2:10 (orange) to 10:10 (green) extended the shelf life of tocopherol by roughly a threefold increase. This profound protective effect aligns with previous literature indicating that food antioxidants can interact synergistically, effectively regenerating one another and enhancing overall oxidative stability (Bensid et al. 2022).

Notably, at both initial concentration ratios in Figure 2, tocopherol degrades in a distinct biphasic pattern: a prolonged, initial period of slow decay is abruptly followed by accelerated, rapid loss. This suggests two distinct chemical regimes operating during storage: an initial “saturation” phase where abundant myricetin provides highly effective mutualistic protection, followed by a rapid degradation phase as myricetin is consumed and its synergistic protective effect collapses.

To rigorously analyze the underlying kinetics driving this biphasic behavior, we applied the machine learning‐augmented system developed in Section 2.2. The AI framework successfully discovered that these interactions are governed by saturation kinetics, confirming the visual behavior in Figure 2 (Section 3.1). These data‐driven insights were then used to develop a fully interpretable, analytical mathematical model (Section 3.2).

### 3.1 Deep Learning Uncovers Saturating Mutualistic Interactions

**FIGURE 3:**
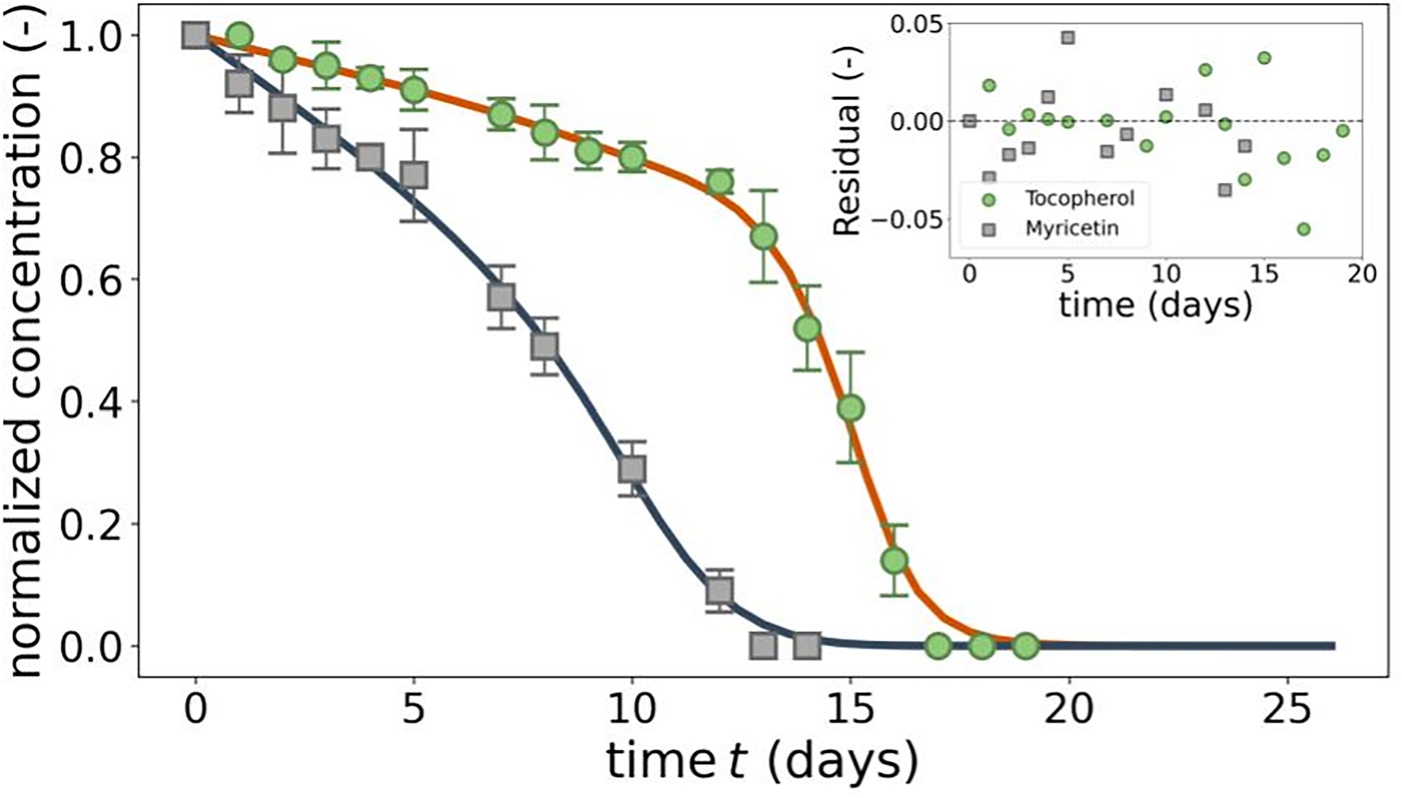
Hybrid model reproduces coupled antioxidant dynamics. Comparison of hybrid model predictions (Equation 1, solid lines) with experimental measurements of tocopherol (circles) and myricetin (squares) for the 2:10 myricetin:tocopherol ratio. Learned linear decay rate coefficients are 𝑎1 = 1.213 (tocopherol) and 𝑎2 = 1.432 (myricetin). Values are presented as normalized, dimensionless concentrations, calculated by dividing the transient concentration by the initial concentration at time zero (10 µM for myricetin and 50 µM for α‐tocopherol). The inset shows the corresponding residuals (observed—predicted) over time for both tocopherol and myricetin, visually confirming the accuracy of the hybrid model and the absence of systematic bias.

The hybrid dynamical system was solved using the sparse, time‐resolved experimental measurements from the 2:10 myricetin:tocopherol ratio. The goal was to let the neural network independently extract the unknown interaction terms, *N1* and *N2*. As shown in Figure 3, the hybrid model accurately reproduced the coupled dynamics of both antioxidants (solid lines), despite being trained on a highly limited dataset (symbols).

To ensure the hybrid model learned the true underlying kinetics, the neural networks were evaluated using a fivefold cross‐validation, with the model iteratively trained on 80% of the data and validated on the remaining 20%. The validations were highly successful, demonstrating excellent generalization across all folds (with an average fivefold validation *R^2^* of 0.937). In addition, the mean squared error (MSE) of the validation sets remained within 6% of the training error. These stable metrics confirm that the neural network architecture successfully avoided overfitting and robustly captured the underlying chemistry of the antioxidant interactions.

#### 3.1.1 Neural Networks Reveal Hyperbolic and Sigmoidal Chemical Interactions

**FIGURE 4:**
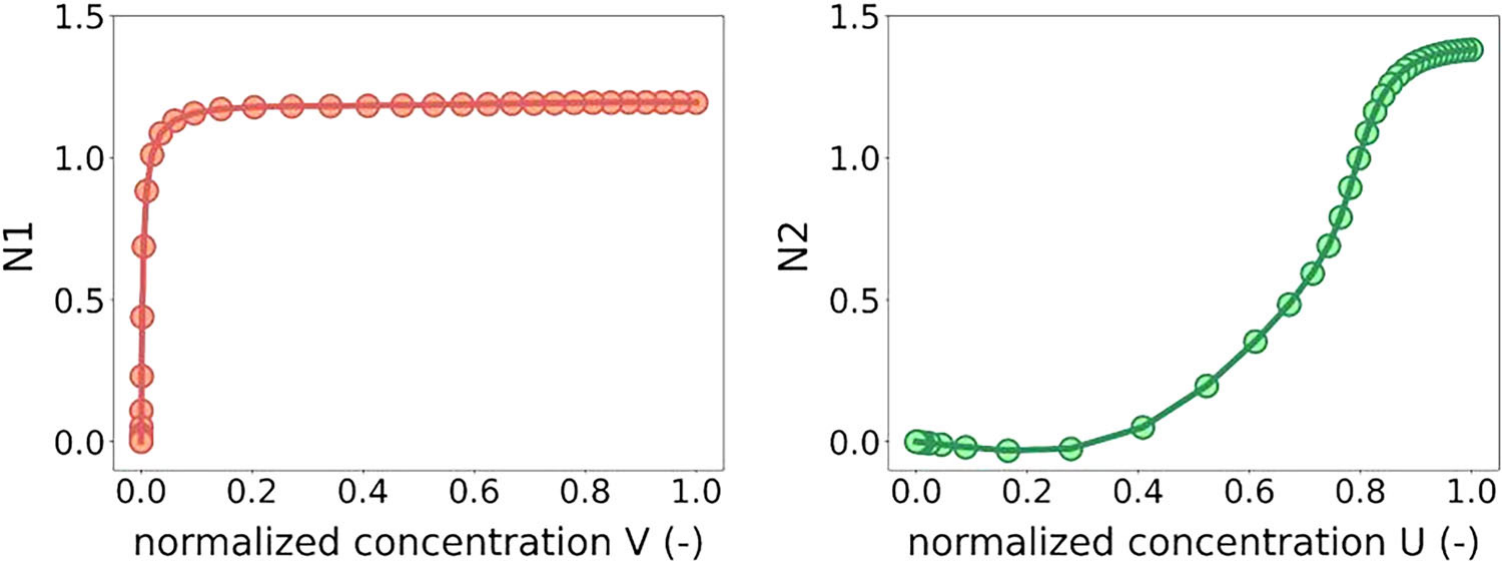
Learned interactions reveal early‐time saturation. Left panel: The neural network 𝑁1 representing the effect of myricetin on tocopherol, shows a hyperbolic dependence on myricetin concentration 𝑣 that quickly saturates. Right panel: The neural network 𝑁2 representing the effect of tocopherol on myricetin, shows a sigmoidal dependence on tocopherol concentration 𝑢. Note that the reaction evolves from high to low concentrations (right to left) over time.

Plotting the learned neural network terms provides a visualization of the exact nature of the antioxidant synergy (Figure 4). The term *N1* quantifies the protective influence of myricetin (*v*) on tocopherol degradation, while *N2* quantifies the reciprocal influence of tocopherol (*u*) on myricetin. Crucially, both networks yielded largely positive values. In the context of Equation 1, these positive values represent a mutualistic protection mechanism, where the presence of one antioxidant reduces the (negative) degradation rate of the other, enhancing the overall stability of the system.

Furthermore, the machine learning framework revealed that both interactions exhibit early‐time saturation kinetics. The protective effect of myricetin on tocopherol (Figure 4, left panel) follows a hyperbolic curve: the protection is maximal at the start of the reaction (when myricetin concentration, *v*, is high) and remains near maximum capacity until myricetin is nearly depleted, at which point the protective effect collapses rapidly. Conversely, the protective effect of tocopherol on myricetin (Figure 4, right panel) exhibits a sigmoidal dependence, suggesting a threshold concentration is required to achieve maximal protective efficacy. Because the UDE framework learns the continuous governing differential equations rather than relying on point‐to‐point data interpolation, it is highly robust to variations in data distribution. The physical validity of these interaction shapes is further corroborated by independent validation (in Section 3.2.1), where the same saturation‐type behavior is required by a purely analytical model to extrapolate to unseen experimental conditions.

### 3.2 From Black‐Box AI to a Fully Analytical Kinetic Model

While neural networks are excellent at discovering hidden patterns, they remain black boxes that are difficult for food formulators to interpret chemically. Having used the UDE framework to uncover the mutualistic, saturation‐type nature of the antioxidant interactions, the next step was to replace the neural networks with a fully transparent, analytical kinetic model.

The interaction shapes discovered by the UDE (hyperbolic and sigmoidal saturation) are classical hallmarks of Hill‐type kinetics. Widely employed in biochemistry and pharmacology, the Hill function generalizes the Michaelis–Menten equation to encompass cooperative saturation behaviors (Alon 2019; Hill 1910). The neural network outputs were analytically represented using simple Hill functions (*H1* and *H2*):

(2a) $$H_{1}\left(v\right)=b_{1}\;v^{n1}\;/\left(v^{n1}+k_{1}\right),$$

(2b) $$H_{2}\left(u\right)=b_{2}\;u^{n2}\;/\left(u^{n2}+k_{2}\right).$$

**FIGURE 5:**
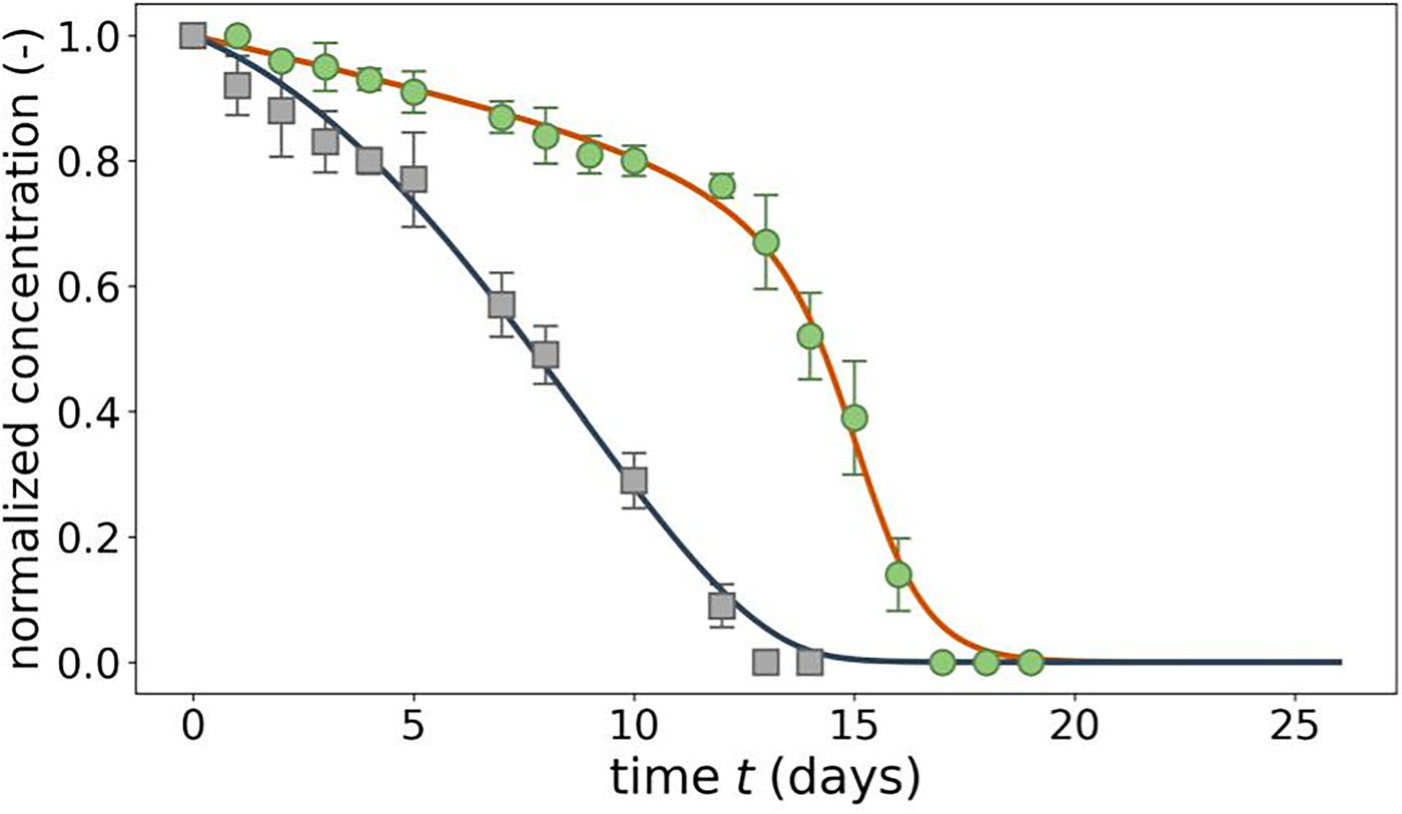
Hill model reproduces coupled antioxidant dynamics. Comparison of the Hill model predictions (Equation 3, solid lines) with experimental data for tocopherol (circles) and myricetin (squares) for the 2:10 myricetin:tocopherol ratio (R^2^ = 0.998). Fitted Hill function parameters are *𝐩𝟏* = (1.207, 0.00887, 0.872) and *𝐩𝟐* = (1.487, 0.0548, 8.429) (see Equation 2). Values are presented as dimensionless normalized concentrations, calculated by dividing the transient concentration by the initial concentration at time zero (10 µM for myricetin and 50 µM for α‐tocopherol).

In Equation 2, *b1* and *b2* denote the maximum cooperation, and *n1*, *n2* denotes a positive Hill coefficient (Alon 2019). By substituting these Hill functions back into our original differential equations (that is replacing *N1* and *N2* in Equation 1), the system is transformed from a ML‐driven model into a traditional, fully analytical system of kinetic differential equations

(3a) $$du/dt=-a_{1}\;u+H_{1}\left(v,p_{1}\right)\;u$$

(3b) $$dv/dt=-a_{2}\;v+H_{2}\left(u,p_{2}\right)\;v$$

where, *pi* = (*bi*, *ki*, *ni*), *i* = 1, 2 represents the vector of parameters of the Hill function. This analytical mathematical model successfully reproduces the observed biphasic antioxidant behavior (Figure 5). Because the Hill functions are mathematically well‐suited to represent the hyperbolic and sigmoidal saturation shapes discovered by the neural networks, they are able to reproduce the learned interaction terms with high accuracy. Consequently, substituting these analytical functions back into the kinetic model naturally yields overall concentration trajectories (Figure 5) that are very close to those generated by the original hybrid model. This confirms that no predictive power was lost when translating the “black‐box” neural networks into mechanistically appropriate analytical equations.

#### 3.2.1 The Analytical Model Successfully Extrapolates to Unseen Formulations

A major limitation of traditional, purely data‐driven machine learning is its inability to extrapolate; purely ML models often exhibit limited extrapolation reliability outside their training domain. In this work, however, the ML model was translated into an analytical system. To assess the true predictive power of the now analytical Hill model, its ability to predict the degradation dynamics of the 10:10 myricetin:tocopherol ratio (an unseen dataset during model discovery) was tested.

**FIGURE 6:**
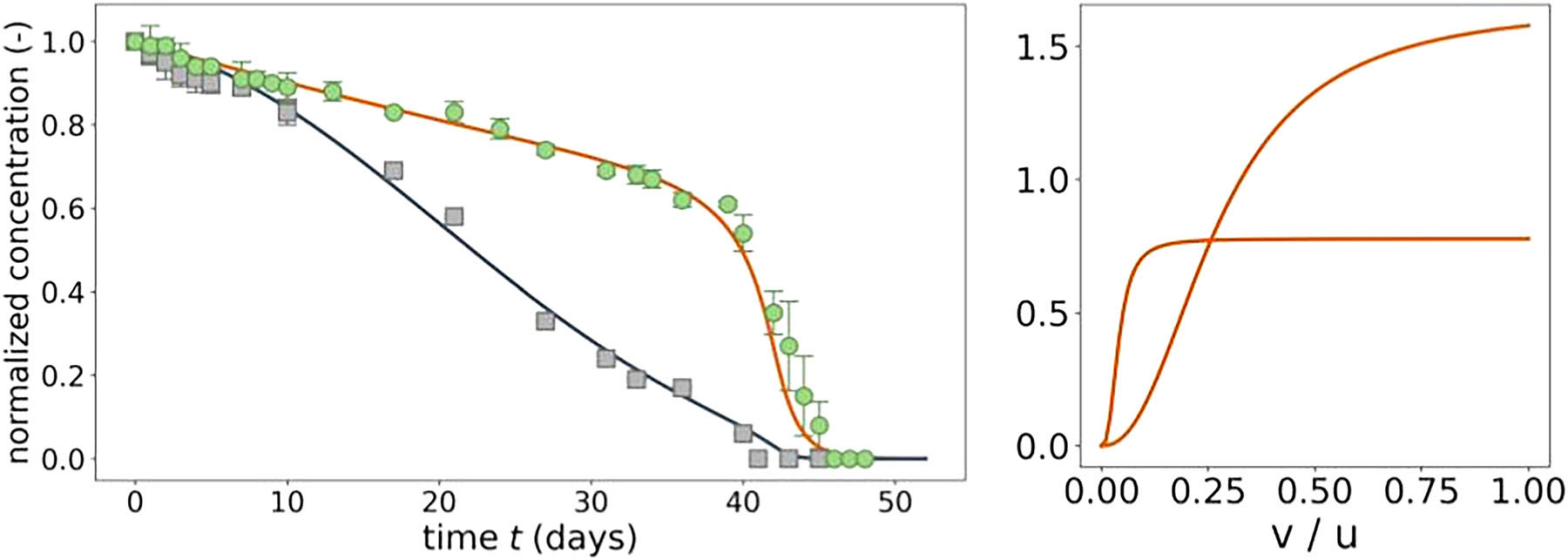
Hill model predicts dynamics of challenging untested data. (Left panel) Comparison of the Hill model predictions (Equation 3, solid lines) with experimental data for tocopherol (circles) and myricetin (squares) for the 10:10 myricetin:tocopherol ratio (*R^2^* = 0.978). This dataset was not used for model discovery. (Right panel) Magnitude of the corresponding hyperbolic and sigmoidal interaction function *H*1(𝑣) and *H*2(𝑢), respectively. The fitted Hill function parameters are p1 = (0.777, 0.000201, 2.648) (tocopherol), and p2 = (1.657, 0.0499, 2.305) (myricetin) (see Equation 2). Values are presented as dimensionless normalized concentrations, calculated by dividing the transient concentration by the initial concentration at time zero (50 µM for both α‐tocopherol and myricetin).

This is a highly challenging test, representing a fivefold increase in myricetin concentration compared to the training data, and a substantial extension of tocopherol shelf life from 17 to 46 days, a threefold increase. Remarkably, the analytical Hill model showed excellent agreement with the experimental degradation dynamics of the 10:10 ratio (Figure 6, left panel). This large, successful extrapolation shows that the Hill model captured the meaningful interaction dynamics.

This generalization capability is not necessarily limited to the specific antioxidant system studied here. Because the UDE framework learns interaction terms directly from time‐resolved experimental data while preserving the governing kinetic structure, the methodology could potentially be extended to other complex food systems involving coupled degradation or reaction dynamics, including antioxidant mixtures, enzymatic reactions, and microbial processes.

From an industrial perspective, this hybrid framework could potentially serve as a data‐efficient in silico formulation tool. Rather than relying exclusively on lengthy experimental shelf‐life studies, a limited number of targeted experiments could be used to train the hybrid model, after which the resulting analytical equations may help evaluate virtual formulation scenarios and identify promising candidates for further experimental validation.

#### 3.2.2 Mechanistic Origin of the Biphasic Tocopherol Profile

Given this robust predictive capability, the analytical model allows us to explain the mechanistic origin of the biphasic tocopherol profile. The separation of timescales observed in Figure 2 is driven by a qualitative shift in the effective kinetic order of the interaction term (*H1*) as myricetin is consumed.

At high myricetin concentrations (*v* ≫ *k1*), the system is in an early‐time saturation regime. When v ≫ k1, the interaction term simplifies to a constant *H1 ≈ b1* (see Equation 2):

(4) $$H_{1}=b_{1}\;\;v^{n1}\;/\left(v^{n1}+k_{1}\right)\approx \;b_{1},$$

Thus, exhibiting the approximately zero‐order kinetics with respect to myricetin. At high myricetin concentrations, the protection is maximized and concentration independent.

However, as myricetin is depleted and falls below a critical threshold (*v* ≪ *k1*), the system transitions to a late‐time regime. With *v* ≪ *k1* now the interaction term becomes proportional to the concentration:

(5) $$H_{1}=b_{1}\;v^{n1}\;/\left(v^{n1}+k_{1}\right)\;\approx \;\left(b_{1}\;/k_{1}\right)\;v^{n1},$$

Thus, shifting to *n*1‐order kinetics. This sudden mathematical shift from zero‐order (*H1* ∼ *b1*, constant protection) to n1‐order (*H1* ∼ *v^n1^*, rapidly decaying protection).

The behavior is clearly illustrated in Figure 6 (right panel), where the interaction *H1* transitions from its saturation horizontal asymptote (the zero‐order regime at high *v*) to a region of near‐vertical dependence (the 𝑛1‐order regime at low *v*), and helps explain the transition from the initial slow decay to the subsequent rapid degradation of tocopherol observed in the experiments (Figure 2).

## 4 Conclusion

The results indicate that the synergistic interaction between α‐tocopherol and myricetin in oil is governed by a mutualistic, saturation‐type mechanism that strongly influences oxidative stability. This interaction provides a mechanistic explanation for the observed biphasic degradation profile of tocopherol, which is driven by a qualitative shift in the effective kinetic order as myricetin is consumed. During the early stages of oxidation, when myricetin concentrations remain sufficiently high, the system resides in a quasi‐zero‐order regime characterized by maximal and near constant protection. However, once myricetin concentrations decline below a critical threshold, the system shifts to a higher‐order (*n*1‐order) kinetic regime, leading to a rapid loss of antioxidant protection and accelerated antioxidant loss. The success of the hybrid artificial intelligence framework to resolve these dynamics is demonstrated by the resulting Hill analytical model, which showed strong predictive performance when extrapolated to an unseen formulation. Notably, the model accurately captured a threefold extension in tocopherol shelf life produced by a fivefold increase in myricetin concentration. These findings establish a practical strategy for integrating agnostic machine learning with mechanistic food chemistry to generate robust and interpretable models from sparse experimental datasets. For the food industry, the implications are substantial: this methodology offers a data‐efficient digital tool that can be operationalized to optimize complex antioxidant formulations, generalize shelf‐life predictions in silico across related food systems without exhaustive empirical testing, and accelerate the rational design of food preservation strategies to reduce waste.

## Author Contributions

**Jiakai Lu**: investigation, formal analysis, writing – original draft. **Sankaran Iyer**: software, visualization. **Ipek Bayram**: investigation. **Eric A. Decker**: investigation. **Shyamvanshikumar P. Singh**: software, validation. **Carlos M. Corvalan**: conceptualization, supervision, formal analysis.

## Conflicts of Interest

The authors declare no conflicts of interest.

## Data Availability

Data are available from the corresponding author upon reasonable request.
